# Immunodiagnostic Biomarkers for Hepatocellular Carcinoma (HCC): The First Step in Detection and Treatment

**DOI:** 10.3390/ijms22116139

**Published:** 2021-06-07

**Authors:** Mengtao Xing, Xinzhi Wang, Robert A. Kiken, Ling He, Jian-Ying Zhang

**Affiliations:** 1 Department of Pharmacology, China Pharmaceutical University, Nanjing 210009, China; mxing@miners.utep.edu; 2 Department of Biological Sciences & NIH-Sponsored Border Biomedical Research Center, The University of Texas at El Paso, El Paso, TX 79968, USA; xwang7@utep.edu (X.W.); rkirken@utep.edu (R.A.K.); 3 Jiangsu Key Laboratory of Drug Screening, Jiangsu Center for Pharmacodynamics Research and Evaluation, China Pharmaceutical University, Nanjing 210009, China

**Keywords:** hepatocellular carcinoma, immunodiagnostic marker, autoantibodies to tumor-associated antigens, exosomes, immunotherapy

## Abstract

Hepatocellular carcinoma (HCC) exerts huge effects on the health burden of the world because of its high mortality and poor prognosis. HCC is often clinically detected late in patients. If HCC could be detected and treated earlier, the survival rate of patients will be greatly improved. Therefore, identifying specific biomarkers is urgent and important for HCC. The liver is also recognized as an immune organ. The occurrence of HCC is related to exacerbation of immune tolerance and/or immunosurveillance escape. The host immune system plays an important role in the recognition and targeting of tumor cells in cancer immunotherapy, as can be seen from the clinical success of immune checkpoint inhibitors and chimeric antigen receptor (CAR) T cells. Thus, there is a pressing medical need to discover immunodiagnostic biomarkers specific to HCC for understanding the pathological mechanisms of HCC, especially for immunotherapy targets. We have reviewed the existing literature to summarize the immunodiagnostic markers of HCC, including autoantibodies against tumor-associated antigens (TAAs) and exosomes, to provide new insights into HCC and early detection of this deadly cancer.

## 1. Introduction

Hepatocellular carcinoma (HCC) is the most common type of primary liver cancer, which constitutes around 80% of cases [1]. It poses a huge health risk and is the sixth most diagnosed cancer worldwide and the fourth leading cause in 2018. [2]. Contributing factor to its poor prognosis is the absence of obvious symptoms during the early stages of HCC. Consequently, greater than 60% of patients with HCC are diagnosed in advanced stages [3], leading to an extremely low overall five-year survival rate (less than 16%) [4]. However, there are many effective curable therapies for early HCC, making a good prognosis for early patients. For example, when a patient is diagnosed with HCC in Barcelona-based clinical liver cancer (BCLC) stage 0 and A, the 5-year survival rate is higher than 93% through surgical intervention therapies [5]. Thus, novel biomarkers for early HCC detection significantly impact curative treatment regimens. This exciting development of HCC biomarkers may encourage the use of more effective and novel chemoprevention strategies for people at high risk for HCC, such as HBV-infected individuals.

The ideal HCC biomarker is one that can be widely employed during a rapid and inexpensive screening process, and even asymptomatic patients can be diagnosed by trained clinicians. Generally, clinically useful biomarkers can achieve at least 90% sensitivity and specificity levels, and are non-invasive and cost-effective to be widely used [6]. Therefore, the most desirable biomarkers are tumor-specific and readily detectable in body fluids, for example, serum, plasma, and bile. Over the past decades, serum α-fetoprotein (AFP) has been used for HCC early detection, but elevated levels of AFP has also been shown to have predictive power in other disease, such as acute viral hepatitis A (AHA) [7]. Since AFP is generally less sensitive to HCC detection (20–65%), serum AFP is no longer in the recommended guidelines [8]. The use of ultrasonography has gained some acceptance, however its effectiveness depends on the operator and has limited ability to clearly differentiate between HCC and benign nodules [9]. Dual-phase computed tomography (CT) scan, and magnetic resonance imaging (MRI) are more effective when the nodules are larger than 1–2 cm [10]. Ineffective diagnostic biomarkers result in an inadequate diagnosis of a large number of HCC patients and false positives in patients with non-tumor liver disease, such as hepatitis and cirrhosis. Because of its dual blood supply from both the systemic circulation and the portal vein, the liver is an important immune organ. Under physiologic conditions, it exerts a protective effect by promoting immune tolerance [11]. Since HCC has been shown as an inflammatory tumor [12], it is necessary to review early immunospecific biomarkers to immunologically diagnose HCC. The use of serum autoantibodies to tumor-associated antigens (TAAs) as serological cancer biomarkers is growing in popularity. The source of this interest is based on the cognition that anti-TAAs antibodies are "indicators" of the tumorigenesis associated with molecular events.

## 2. Tumor-Associated Antigens (TAAs) and Anti-TAAs Autoantibodies

As early as the 1960s, Robert W. Baldwin showed that the human immune system can produce autoantibodies in the early stage of cancer development [13]. The normal proteins of the human body do not have immunogenicity because of self-tolerance. A widely accepted view is that mutations, ectopic or via recombination, can occur early in the development and progression of tumor cells [14]. During tumorigenesis and progression, altered protein expression levels, an environment of chronic inflammation, protein structure changes, and cell death mechanisms will trigger specific proteins, called TAAs, promoting the host’s immune responses. Ultimately, the autoantibodies against TAAs, which can be regarded as "sensors" or "reporters" that indicate abnormalities or dysregulation of cellular mechanisms during tumorigenesis, are detectable in the serum. Therefore, these specific autoantibodies can be used as potential biomarkers in early cancer diagnoses, a detector of treatment results, and even an indicator of cancer prognosis [15,16].

**Figure 1:**
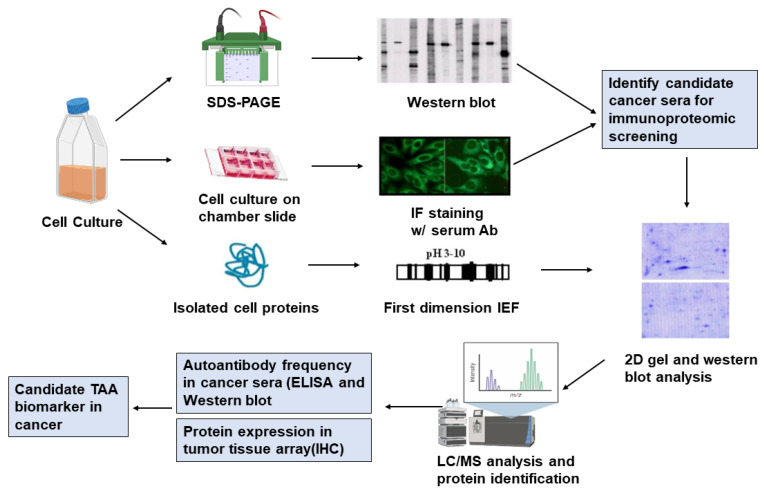
Using immunoproteomic approach for identification and validation of TAAs. Firstly, the sera from HCC patients with high titer fluorescently stained were selected out with western blotting analysis and indirect immunofluorescence by using cultured tissue cells as antigen. Candidates of TAA were then separated using 2D-SDS-PAGE and analyzed by mass spectrometry. Finally, multiple methods, such as enzyme-linked immunosorbent assay (ELISA), Western blotting and immunohistochemistry (IHC), and tissue arrays, are used to validate identified potential tumor-associated antigen-antibody systems. These validated TAAs can be used to form TAA arrays for the immunodiagnosing early-stage cancers.

Cancer-specific autoantibodies, usually present at very low titers or absent in non-cancer individuals, are produced by the self-immune system in response to TAAs. Modern biological methods have been able to detect low concentrations of antibodies that cannot be detected by traditional in vitro testing [17]. In addition, antibodies are not only stable in vivo, whose survival time in the circulation of cancer patients is usually up to 30 days, but also more stable in vitro than other biomarkers. Based on these unique advantages, anti-TAAs autoantibodies are ideal biomarkers for the early detection of tumorigenesis [18]. The shortcoming of autoantibodies against single TAA in diagnosis is the low frequency (10–20 %) in HCC sera [15]. For this disadvantage, our group found that this drawback can be overcome by using a carefully selected TAA microarray. But for different types of cancers, may need to design different TAA arrays to achieve the needed sensitivity and specificity to make this method a reliable reference for clinicians’ tumor diagnosis (Figure 1). We designed a microarray for HCC diagnosis that includes various TAAs, such as tumor suppressors p53, Ink4a/p16, NPM1/B23, CAPERα/HCC1.4, and oncoproteins, including IMP1, IMP2/p62, IMP3/Koc, CIP2A/p90, RalA, c-Myc, survivin, cyclin B1, 14-3-3ζ, and MDM2. The sensitivity and specificity of 14 TAAs for immunological diagnosis of HCC were 69.7% and 83.0%, respectively [19]. It is noteworthy that 43.8% of HCC patients identified by this microarray had normal serum AFP levels [19]. In a recent study, a panel of 12 TAAs was used to establish a predictive value based on the sera from 160 HCC patients and 90 normal controls and then successfully used to diagnose HCC in 16 of 17 patients who had no earlier clinical information prior to diagnosis [20]. In a series of studies, it was shown that the study of anti-TAAs panels as biomarkers acts as a potential early detection method for HCC.

The nature of autoantibodies against different types of TAAs is different. For tumor suppressors, the most likely reason is mutation. TP53, as a famous tumor suppressor gene, frequently mutated in HCC patients (~30%), and its autoantibodies can be detected in 20% of HCC patients. Different from this, the autoantibodies against the proteins encoded by oncogenes are usually produced, due to the protein overexpression [21,22]. Oncoproteins IMP1, IMP2/p62, and IMP3/Koc belong to the IGF-II mRNA binding protein (IMPs) family. They are highly conserved and share a similar structure: The identity of the overall amino acid sequences approximately is 60-80%; all three members have six characteristic RNA binding modules, including two N-terminal RNA recognition motifs (RRMs), and four heteronuclear ribonucleoproteins (hnRNP) K-homology (KH) domains in their C-terminal regions [23]. IMPs are mainly expressed during embryonic development and almost disappear completely in adult tissues. Nevertheless, they are again highly expressed in a variety of cancer tissues, so they are considered oncofetal proteins [24,25]. In 1999, we first identified IMP2 as a TAA in the sera of HCC patients. The autoantibodies against IMP2 can be detected in the early stage of HCC and approximately 21% of HCC patients [19,23]. In subsequent work, we found that the expression of IMP2 was elevated in both mRNA and protein levels in HCC. Overexpression of IMP2 can enhance the signal of the Wnt/β-Catenin pathway in HCC, induce the genomic instability and improve the migration ability of HCC cells [26,27]. In addition, IMP1 and IMP3 play important roles in the development of HCC [28,29]. We also found a high frequency of autoantibodies of IMP1 and IMP3 existed in the sera of HCC patients [19]. The IGF2 mRNA is one of the common targets of IMPs, which is a fetal growth factor and has a similar structure and function to IGF1 [30]. IGF2 is not expressed in healthy adults. However, the frequency of various cancers occurred more frequently in IGF2 transgenic mice [31]. In human HCC, overexpression of IGF2 is associated with fetal malformations and a variety of cancers [32]. Under the regulation of IMPs, the expression of IGF2 in HCC was significantly increased [33]. Meanwhile, IGF-2 can also be recognized as a cofactor or a second signal for transformation in SV40 oncogene-induced tumorigenesis [34]. In summary, the TAAs like IMPs, can play an important role in carcinogenesis by modulating its downstream signaling, and their autoantibodies could be regarded as valuable biomarkers in the early diagnosis of HCC.

In Table 1, a comprehensive list of autoantibodies used in HCC diagnosis which have been reported since 1997.

## 3. HCC-Derived Exosome

Exosomes are membrane-bound extracellular vesicles that are 40–100 nm in size. Under normal and pathological conditions, almost all cell types secrete exosomes. Exosomes existing in various body fluids (blood, urine, ascites, and et al.) cargoes a wide range of biomolecules, including lipids, messenger RNAs (mRNAs), microRNAs (miRNAs), long non-coding RNAs (lncRNAs), and proteins [82]. These features of exosomes could act as potential diagnostic and monitoring biomarkers for cancer [83]. (1) The amount and content of exosomes reflect the actual conditions of their original cells with high sensitivity, because exosomes can be immune-isolated using antibodies against tissue-specific proteins on the cell membrane surface. (2) The stable structure of lipid bilayer protects exosomal cargoes from damage by widespread RNases and proteases in circulation, thus exosomes can keep their cargos intact and have high fidelity and consistency. (3) Release of exosomes into biological fluids provides a non-invasive way to determine patients with tumors. (4) In contrast to the hardly detectable signal in biological fluids, exosomes comprise abundant specific cancer proteins, non-coding nuclear acids, and other markers, which facilitates the recognition of low abundance nucleic acids or proteins while increasing the signal-to-noise ratio during detection [84]. (5) The comparatively uncomplicated structure of exosomes reduces the complexity of the identification. Moreover, they may have circulating biomarkers with more accuracy, which can be a benefit for the early diagnosis of tumors. (6) Exosome analysis can directly generate details on disease lineage, staging, relapse, and drug reaction [85]. Therefore, exosomes might represent a “liquid biopsy” for malignancies.

Extensive researches have investigated serum exosomal miRNAs as potential biomarkers. As shown in Table 2, two exosome miRNAs, miR-21, and miR-122, appear repeatedly. Serum miR-21 is an important indicator used to independently assess relapse and is revealed to have more sensitivity than AFP in detecting HCC [86]. In comparison to normal individuals or patients with chronic hepatitis B (CHB), patients with HCC showed increased exosomal miR-21 levels, which is related to LC and advanced tumor stage. Moreover, compared with whole serum free of exosomes, the exosomal miR-21 level is obviously more elevated, which shows higher diagnostic sensitivity [87]. The above studies suggest that miRNAs in exosomes of circulation, like miR-21, can be used to predict HCC risk and detect HCC early. However, a report has also shown that miR-21 level in serum of HCC patients is decreased in comparison to chronic hepatitis patients [88]. Similarly, there is no increase in miR-21 levels of HBV-related HCC patients. Therefore, circulating miR-21 levels cannot distinguish patients with cirrhosis from those with HCC [89]. Identification techniques of miRNA, patient selection, and shortage of general internal controls of miRNA may contribute to this difference. Liver-specific miR-122 plays a suppressive role in the HCC progress by binding to target genes related to cell proliferation, migration, differentiation, apoptosis, and angiogenesis in HCC [90]. MiR-122 not only has diagnostic value for HCC, but also has been shown to have relation to the fibrosis of the liver [9,91], and viral replication rate [92]. Circulating levels of miR-122 are related to liver damage, as well as high ALT levels [93]. If the levels of exosomal miRNA match those in the parental cells, the result may suggest that exosomal miR-122 reflect residual liver function and capacity [94].

Many studies have been performed to investigate the possibility of exosomes as markers for different stages of HCC. Compared with serum circulating mi-RNA levels, serum exosomal mi-RNA levels can better distinguish HCC from CHB or LC. It has been revealed that the circulating levels of exosomal miR-18a, miR-221, miR-222, and miR-224 in HCC patients are significantly increased compared to patients of CHB or liver cirrhosis (LC) [95]. However, the serum levels of exosomal miR-101, miR-106b, miR-122, and miR-195 of HCC patients are reduced compared to CHB patients [95]. The prognosis for HCC patients remains unsatisfactory because of the great prevalence of postoperative relapse and cancer proliferation. MiR-125b in exosomes of serum is served as a biomarker in predicting relapse and survival in HCC patients after surgery [96]. In addition, exosomal lncRNA-ATB acts as a non-invasive predictor of HCC prognosis, which is independent of age, gender, the existence of LC, or cause [97]. It has been demonstrated that lncRNA-ATB promotes ZEB1 and ZEB2, thereby cause EMT, attack from cancer cells, and spread of cancer [98]. The serum level of exosomal miR-718 of recurrent HCC patients after LT is obviously reduced compared with patients free of recurrence, which has a positive relation to poor prognosis and aggressiveness of HCC patients [99]. Recently, accumulating evidence has identified that exosomes select particular miRNAs into their packages dependent on conditions [100]. The miRNA expression profiling in exosomes of the HCC cell line SMMC-7721, is different from the parental cells, suggesting that exosomes act as regulators of miRNAs in cells [101]. Exosomes can also cargo proteins with significant diagnostic or prognostic potential. Proteomics identification has found differences in serum exosomes of cholangiocarcinoma (CCA), primary sclerosing cholangitis (PSC), HCC, and normal controls. Serum exosomal galectin-3-binding protein (LG3BP) and polymeric immune receptor (PIGR) reveal more sensitivity in the detection of HCC than AFP. Both are oncogenic proteins, promoting the progression of HCC, malignant transformation, aggression, and proliferation [102]. LG3BP is also considered a biomarker for the poor prognosis of HCC [103].

Exosomes are a kind of non-invasive, sensitive, and specific biomarkers that serve as diagnostic, prognostic, and predictive markers for HCC (Table 2). Unlike conventional cancer biomarkers, including carcinoembryonic antigen and AFP, exosomal miRNAs can transfer their functions to target cells and modulate cell signaling [104]. Generally, tumor cells produce an increasing number of exosomes than normal cells [105]. Tumor-derived exosomes contribute to an appropriate microenvironment for tumor cells to proliferate, reduce in effectiveness of the drug, generate blood vessels, invasion, regulate the immune system, and form metastatic niche before metastasis [106]. In particular, exosomes are essential for cell-cell communication by transmitting information from tumor cells to nearby or distant cells. In addition, exosomes can induce immune responses and modulate the immune system [107]. Overcoming the resistance of HCC to chemotherapy is also a challenge. Exosomes display a range of HCC antigens [108]. Cancer-derived exosomes have been shown to trigger a stronger immune response mediated by dendritic cells (DC) rather than cell lysates, and ameliorate the tumor microenvironment (TEM) of HCC [109]. Exosomes secreted by AFP-expressing DCs (DEXAFP) induce intense immune responses based on antigen specificity, which lead to significantly delayed cancer development and increased survival rates in murine models of HCC. Therefore, DEXAFP may provide a promising vaccine without cells for immunotherapy of HCC [110]. Moreover, HCC cell-derived exosomes enhance drug resistance of sorafenib in vitro. However, AMSC-derived exosomes are effective carriers of miRNA-122 and can increase the chemosensitivity of HCC [111]. These results provide potential immunotherapies for the improvement of therapeutic efficacy.

## 4. Other Immunodiagnostic Biomarkers

Squamous cell carcinoma antigen (SCCA) is a kind of serine protease inhibitor known as serpins [124]. Under physiological conditions, SCCA is expressed by the stratum granulosum of normal squamous epithelium. Under pathological conditions, it is found in carcinoma cells, such as lung tumors, cervical tumors, and head and neck tumors [125]. SCCA is absent in normal liver cells, whereas its expression is elevated when liver inflammation occurs [126]. Immunohistochemical results showed that variants of SCCA (SCCA1 and SCCA2) are overexpressed in biopsies specimens of HCC [124]. IgM antibodies in circulation constitute antigen-antibody complexes with specific cancer biomarkers, offers chances for the prediction and treatment of HCC patients [127]. The emergence of the biomarker IgM immune complex appears to be confirmed by a model of cancer immune editing, which recognizes natural IgMs as participants of great significance in innate immunity [128]. IgM in circulation identifies new epitopes on the surface of cancer cells and promotes the phagocytosis and clearance of transformed cells by dendritic cells and macrophages. The mechanism reveals a possible host defense that attempts to exert selective pressure on newborn neoplastic cells to fight cancer growth [129]. Compared with LC and control groups, elevated levels of SCCA-IgM immune complexes can be detected in the serum of cirrhosis and HCC patient and assessing SCCA-IgM levels have better diagnostic value than determining the corresponding single molecule [126,130,131]. A significantly positive correlation exists between SCCA-IgM and AFP [132]. The diagnostic accurateness of serum SCCA-IgM is greater than that of AFP, due to its higher AUC, sensitivity, and specificity, which distinguish HCC patients from cirrhosis patients [133]. The level of SCCA-IgM decreases after treatment, suggesting that SCCA-IgM plays a role in the prognostic prediction of HCC. Reduced levels of SCCA–IgM may relate to a decrease in the innate immunity and/or reduced release activity of tumor cells in patients tested positive. This data indicates that the reduced concentration or activity of SCCA in the liver may reflect the decreased invasion, proliferation, and anti-apoptotic properties of cancer cells [134]. Elevated serum SCCA-IgM levels of HCC patients have also been reported to have a relation to the decreased survival, suggesting that serum SCCA–IgM levels may make a significant contribution to the prediction of HCC therapy response [130,135]. 

In addition, an oncofetal protein, Glypican-3 (GPC3), is regarded as a potential biomarker in serum recently. It is a member of the Glypican family and is a heparan sulfate proteoglycan located on the outer surface of cell membranes [136]. GPC3 is expressed in the liver of the fetus, whereas it cannot be detected in the liver of healthy adults [137]. In contrast, GPC3 is overexpressed not only at the gene, but also protein levels in HCC patients. It has been demonstrated in a study that GPC3 mRNA is overexpressed in 55.7-100% of HCC tissues [138]. The same conclusions have also been shown in hepatitis C virus (HCV) infected HCC patients [139]. At the protein level, GPC3 is highly expressed in more than 70% of HCC patients by immunohistochemical staining (21/29), but not expressed in healthy or benign hepatic lesions, cirrhosis, hepatitis, or healthy adult tissues (0/38) [140]. More importantly, research has revealed that GPC3 levels are increased not only in liver cancer tissues, but also in serum. GPC3 can be cleaved and released from the cell surface into the serum. At the same time, GPC3 autoantibodies and GPC3-specific cytotoxic T lymphocytes (CTL) can be detected in the blood of HCC patients [141]. Therefore, the levels of serum GPC3, its autoantibodies, and even specific immune cells can be used as potential diagnostic biomarkers for HCC [142]. In liver cancer diagnosis, AFP is a traditional biomarker, and the expression of GPC3 can distinguish benign nodular liver tumor with AFP negative. Therefore, when diagnosing liver cancer, the combined use of GPC3 and AFP may make the diagnosis more reliable [143]. GPC3 has been the target of molecular imaging for early liver cancer, especially the positron emission tomography imaging technology for GPC3 has matured nowadays [144]. Various immunotherapies targeting GPC3 have also been studied, such as the use of antibodies against anti-GPC3, gene therapy against GPC3, vaccines based on GPC3 peptides, and GPC3-targeted CART therapy, etc. [145].

Chemokines in serum can also be deemed as ideal potential biomarkers for HCC diagnosis. For instance, CXCL13 can cooperate with its receptor CXCR5, attracting T helper cells and to encourage lymphocyte homing of naive B cells [146]. Hence, CXCL13 is also known as B cell attracting chemokine 1 (BCA 1) in humans [147]. Many studies have demonstrated that CXCL13 is deeply involved in the occurrence, development, and metastasis of multiple tumors [148,149]. In HCC, the expression of CXCL13 and CXCR5 are both up-regulated compared to healthy tissues, and even higher in poorly differentiated cancer tissues, which may be related to the harsh tumor microenvironment [150]. Clinical data showed that CXCL13 is elevated in the serum of more than 60% of HCC patients compared to healthy controls. As tumors become larger, the levels of CXCL13 in serum become higher and higher. Correlation analysis showed that serum CXCL13 levels are of high value for the diagnosis and prognosis of HCC [151]. Another clinical research also indicated that the combination of CXCL13 and AFP may potentially increase the sensitivity of AFP to HCC and can be used in the clinical diagnosis of HCC [152]. In the future, we believe that the above-mentioned potential biomarkers will be used in combination with AFP to have a more accurate diagnosis of early HCC.

## 5. Conclusions

The immune system of cancer patients suggests that it can perceive structure, function, internal location of cells, and other changes in cellular participants during tumorigenesis, which can be the first sign of carcinogenesis. Immunodiagnostic markers have been described as the reporters, internal outposts, and immune surveillance of the immune system. Moreover, immunodiagnostic biomarkers, even ones with low sensitivity, have the potential to serve as an important indicator for a tumor-targeted drug. This unexpected assistance by the immune system provides cancer researchers with powerful research tools to unlock important clues to our understanding of oncogenesis and ultimately lead to better cancer treatment strategies.

## Figures and Tables

**Table 1:** TAA as biomarkers in HCC.

Function	Autoantibodies	Control	Refs.
Early diagnosis	↑HCC1, P16, P53, P90, survivin	Healthy individuals	[20]
	↑AFP, AFP-L3, DCP, CENPF	LC, CH, NHS	[35]
	↑SPAG9	LC, CH, NHS	[36,37]
	↑NPM1	LC, CH, SLE, NHS	[16]
	↑14-3-3ζ	LC, CH, NHS	[38]
	↑MDM2	LC, CH, NHS	[39]
	↑CENPF, DDX3, HSPA4, HSPA5, VIM, LMNB1, p53	CH, NHS	[40]
	↑DDX3, eEF2, AIF, hnRNP A2, PBP, TIM	CH, NHS, lung cancer, EC, BC, GC	[41]
Diagnose (complementarydiagnosis)	↑EIF3A	NHS	[42]
	↑SF3B1	NHS	[43]
	↑GAGE-1	LC, HB, NHS	[44]
	↑CAPERα	Prostate cancer, breast cancer	[45]
	↑NY-ESO-1	NHS	[46]
	↑IMP1, IMP2/p62, IMP3/Koc, CIP2A/p90, RalA, c-Myc, survivin, cyclin B1, 14-3-3 ζ, MDM2, p53, CAPERα/HCC1.4, p16, NPM1	LC, CH, NHS	[19]
	↑AFP, Cyclin B1, Gankyrin, p53, NY-ESO-1, RalA, CK8, H-RAS-1, p16, WT1	hepatitis C with either cirrhosis or chronic liver disease, NHS	[47]
	↑ Sui1	LC, CH, NHS	[15]
	↑IMP1, p62, Koc, p53, c-myc, cyclin B1, survivin, p16, RalA, Sui1	LC, CH, NHS	[48]
	↑FASN	NHS	[49]
	↑RalA	LC, CH, NHS	[50]
	↑KRT23, AHSG, RPL17, FTL, DDX41	CH, NHS	[51]
	↑ORP150, aconitate dehydratase, HSP70, protein disulfide-isomerase A3, NDRG1, GLUD1, PA2G4, fumarate hydratase, VDAC1, PEBP, peroxiredoxin	LC, CH, NHS	[52]
	↑EIF3SI, LDHA, RFC2, MCART1	LC, CH, NHS, GC, PC	[53]
	↑IMP1, p62, Koc, p53, c-myc, cyclin B1, survivin, p16	LC, CH, NHS	[54]
	↑HSP70, GAPDH, PRX, Mn-SOD	NHS	[55]
	↑ IMP1, IMP3, p53	LC, CH, NHS	[56]
	↑p16	NHS	[57]
	↑c-myc, p53, cyclin B1, p62, Koc, IMP1, survivin	NHS	[58,59]
	↑p90	CH, AH, HBsAg carriers, NHS	[60]
	↑calreticulin, CK8, and NDPKA, and ATP5F1B,	Other cancers, CH, active SLE, NHS	[61]
	↑p62, CENPF	LC, CH, autoimmune hepatitis	[62]
	↑p62	Asymptomatic HBsAg carrier, AH, CH, NHS	[23]
	↑p53, AFP	chronic liver disease (non-viral/viral liver disease )	[63,64]
	↑cyclin B1	LC, CH, NHS	[65]
Diagnosis, recurrence/metastasis prediction	↑GRP78	LC, CH, NHS SUN449, A549, T24, MOLT-4, KOPN63	[66,67]
Diagnosis/prognostic marker	↑OPN	LC, CH, NHS	[68]
	↑NX-PVKA, DCP	Compare using Child-Pugh class and TNM classification	[69]
	↑p62	EC, GC, large intestine cancer	[70]
Prognostic marker	↑anti-CD25 IgG	NHS	[71]
MVI prediction in HCC	↑HSP 70 and Eno-1	NHS	[72]
Diagnosis of AFP-negative HCC	↑SMP30	LC, CH, NHS	[73]
	↑NPM1, 14-3-3 ζ, MDM2	chronic liver disease, normal human control, AFP-positive HCC cases	[74]
	↑HCC-22-5	LC, CH, NHS, Nasopharynx cancer, lung cancer, gastric-intestine disease	[75]
	↑IMPs	NHS	[76]
Diagnosis of HBV-HCC	↑Ku86	LC, NHS	[77]
	↑hnRNP L	HBV carrier, HBV LC, NHS	[78]
Diagnose of HCV-HCC	↑HSP70, SOD2, PRDX6	HCV-/HCC-, HCV+/HCC-, NHS	[79]
	↑Ku86	LC, NHS	[80]
	↑DHCR24	HBV+ including LC, CH	[81]

AH, acute hepatitis; AIF, apoptosis-inducing factor; AFP, alpha-fetoprotein; AFP-L3, lens culinaris agglutinin-reactive AFP; anti-CENPF, centromere protein F autoantibody; BC, breast cancer; CK8, cytokeratin 8; CH, chronic hepatitis; DCP, des-gamma-carboxyprothrombin; DDX3, DEAD (Asp-Glu-Ala-Asp) box polypeptide 3; DHCR24, 3β-hydroxysterol Δ24-reductase; EC, esophageal cancer; eEf2, eukaryotic translation elongation factor 2; Eno-1, alpha-enolase; FASN, fatty acid synthase; F1-ATP synthase β-subunit; GAGE-1, cancer-testis antigen G antigen 1; GC, gastric carcinoma; GLUD1, glutamate dehydrogenase 1; GRP78, glucose-regulated protein 78; hnRNP A2, heterogeneous nuclear ribonucleoprotein A2; HBV-HCC, hepatitis B virus-related hepatocellular carcinoma; HCV-HCC, hepatitis C virus-related hepatocellular carcinoma; hnRNP L, hnRNP Lheterogeneous nuclear ribonucleoprotein L; LC, liver cirrhosis; MDM2, mouse double minute 2 homolog; MVI, microvascular invasion; NDPKA, nucleoside diphosphate kinase A; NDRG1, N-myc downstream-regulated gene 1; NHS, normal human serum; NPM1, nucleophosmin; OPN, osteopontin; PC, pancreatic carcinoma; PEBP, phosphatidylethanolamine-binding protein; PBP, prostatic binding protein; TIM, triosephosphate isomerase; SLE, systemic lupus erythematosus; SPAG9, sperm-associated antigen 9; VDAC1, voltage-dependent anion-selective channel protein 1. ↑: upregulated biomarkers.

**Table 2:** Exosomes are used as biomarkers of HCC.

Function	Species	Source	Exosome contents	Control	Ref.
Early prediction	Rat	Serum	↑miR-10b, ↑miR-21, ↓miR-122, ↓miR-200a	Normal, degeneration, fibrosis, cirrhosis	[9]
	Human	Serum	↑miR-21	Healthy individuals, CHB patients	[87]
Diagnose	Human	Serum	↑hnRNPH1	Healthy individuals, CHB and LC patients	[112]
	Human	Serum	↑LncRNA HEIH ↓LncRNA HEIH	HCV-induced cirrhosis CHC patients	[113]
	Human	Serum	↓miR-9-3p	Healthy individuals	[114]
	Human	Serum	↑LncRNA-FAL1	Healthy individuals	[115]
	Human	Serum	↑LG3BP, ↑PIGR	Healthy individuals	[102]
	Human	Serum	↑miR-18a, ↑miR-221, ↑miR-222, ↑miR-224 ↓miR-101, ↓miR-106b, ↓miR-122, ↓miR-195	CHB or LC	[95]
	Human	Serum	↑miR-519d, ↑miR-494, ↑miR-21, ↑miR-22	Cirrhotic patients without HCC	[116]
Diagnosis and prognosis prediction	Human	Serum	↑miR-122, ↑miR-125b, ↑miR-145, ↑miR-192, ↑miR-194, ↑miR-29a, ↑miR-17-5p, ↑miR-106a	Healthy individuals	[117]
	HepG2, SMMC7721, SKHEP1, Huh7 cells Human	Cell culture media Serum	↑miR-93	WRL68 cell Healthy individuals	[118]
	MHCC-97H Human	Cell culture media Serum	↑miR-665	MHCC-97L and L02 Healthy individuals	[119]
Diagnosis, clinical staging and recurrence prediction	Human	Serum	↑ENSG00000258332.1, ↑LINC00635	Healthy individuals, CHB and LC patients	[120]
Recurrence/metastasis prediction	Human	Serum	↑miR-103	Recurrence-free survival groups	[121]
	Human	Serum	↑CASC9	Low recurrence survival groups	[122]
Prognosis prediction	Human	Serum	↑miRNA-21, ↑lncRNA-ATB	Two different non-human miRNAs	[97]
	Human	Serum	↓miR-638	Healthy individuals	[123]
	Human	Serum	↓miR-125b	CHB	[96]
Prognosis prediction after LT	Human	Serum	↓miR-718	HCC patients without recurrence	[99]
Prognosis prediction of TACE	Human	Serum	↓miR-122	LC and CH	[94]

CHB, chronic hepatitis B; CHC, chronic hepatitis C; LC, liver cirrhosis; LG3BP, Galectin-3-binding protein; LT, liver transplantation; PIGR, polymeric immune receptor; TACE, transarterial chemoembolization. ↑: upregulated biomarkers ↓: downregulated biomarkers.
